# Supplementary material for: IL-10 Promotes CXCL13 Expression in Macrophages Following Foot-and-Mouth Disease Virus Infection
Source: Int J Mol Sci. 2023 Mar 28;24(7):6322. doi: 10.3390/ijms24076322 (PMC10093876; doi:10.3390/ijms24076322)
Supplement: Supplementary file 1 [file ijms-24-06322-s001.zip › ijms-2197305-supplementary.pdf]

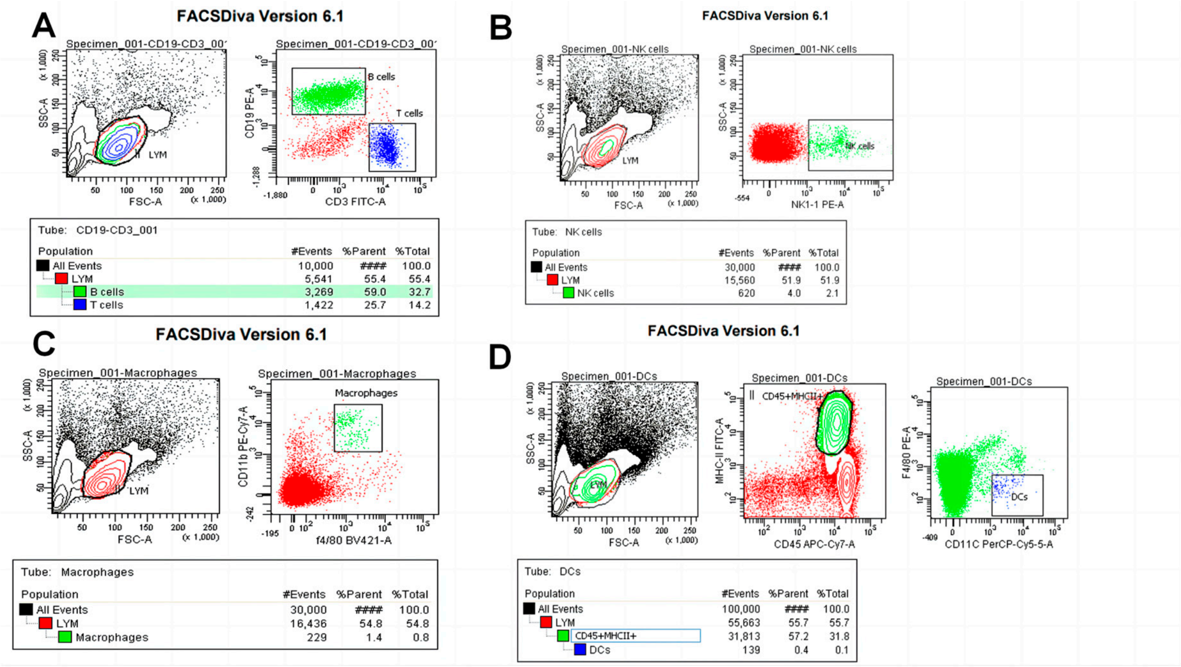

Figure S1: The representative FACS plots of sorted T cells (A), B cells(A), and NK cells (B), macrophages (C), and DCs (D) of mice.

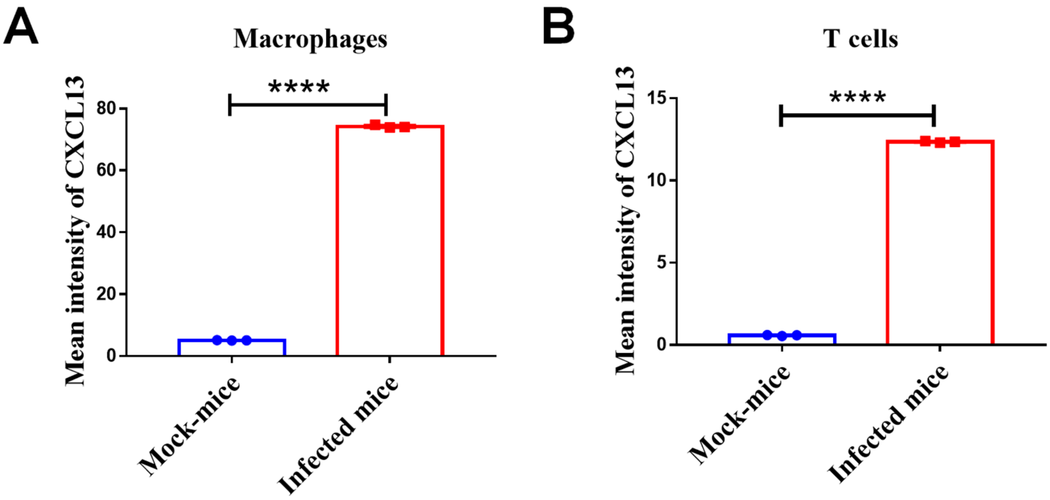

Figure S2: The mean intensity of CXL13 in T cells and macrophages from mock-mice and infected mice.

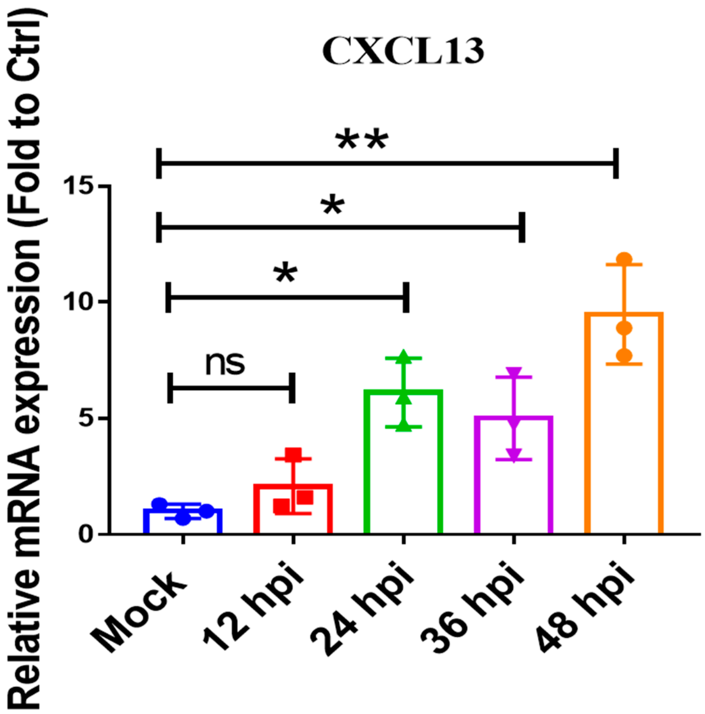

Figure S3: The mRNA expression of CXCL13 in BMDMs at 12, 24, 36, and 48 hpi.

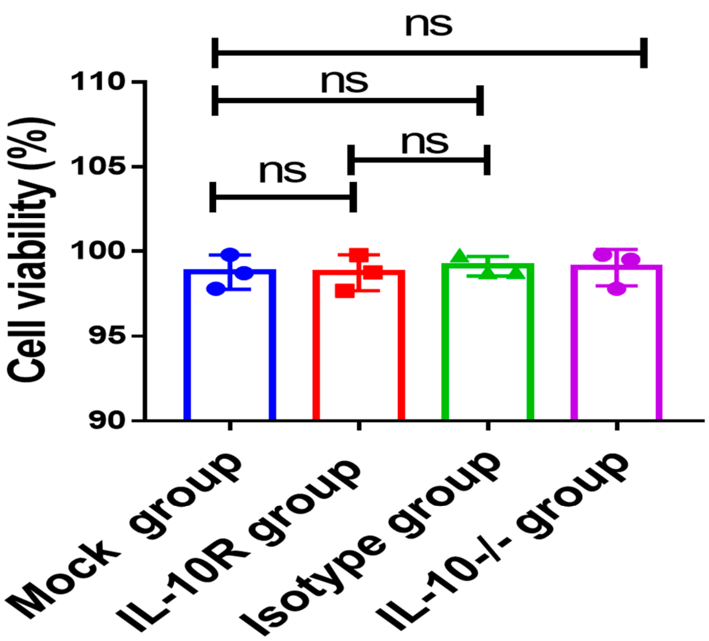

Figure S4: The cell viability of BMDMs, BMDMs with IL-10R blocker treatment, BMDMs with isotype antibody treatment, and IL-10$^{-/-}$ BMDMs.

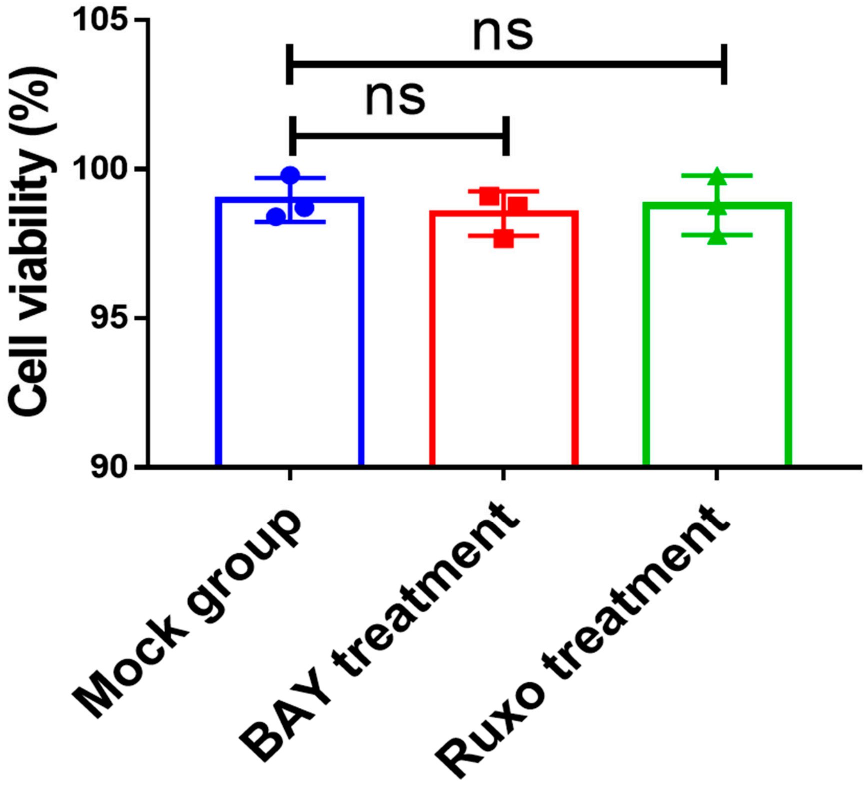

Figure S5: The cell viability of BMDMs, BMDM with BAY treatment, and BMDM with Ruxo treatment.

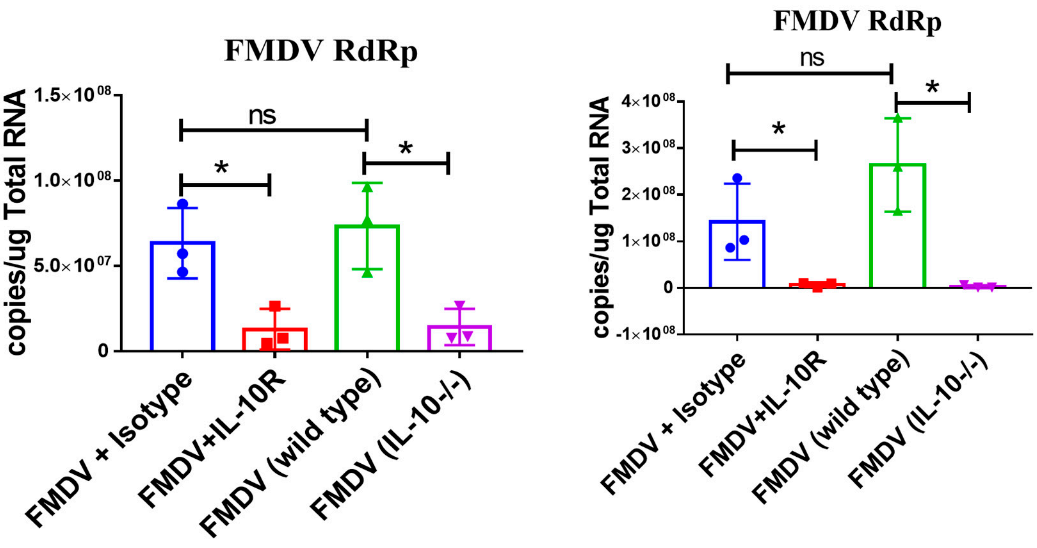

(**A**) (**B**)

Figure S6: The FMDV RNA levels in BMDMs (A) and in sera of mice (B).

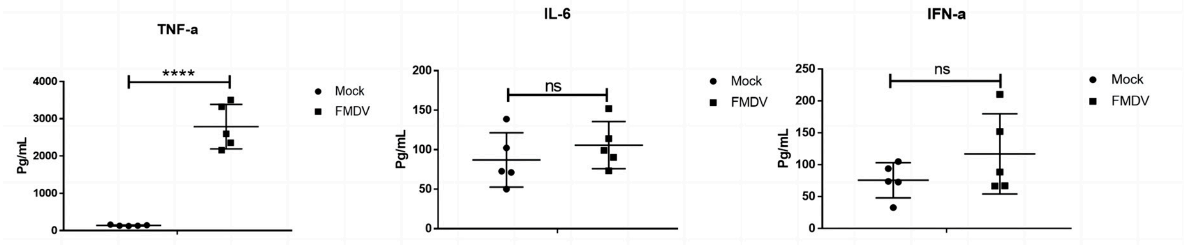

Figure S7: The TNF-α, IL-6, and IFN-a concentrations of the supernatant from BMDMs (Mock) and BMDMs infected with FMDV were measured by ELISA.
